# Identification of Residues in the Heme Domain of Soluble Guanylyl Cyclase that are Important for Basal and Stimulated Catalytic Activity

**DOI:** 10.1371/journal.pone.0026976

**Published:** 2011-11-09

**Authors:** Padmamalini Baskaran, Erin J. Heckler, Focco van den Akker, Annie Beuve

**Affiliations:** 1 Department of Pharmacology and Physiology, New Jersey Medical School, University of Medicine and Dentistry, New Jersey (UMDNJ), Newark, New Jersey, United States of America; 2 Department of Biochemistry, Case Western Reserve University, Cleveland, Ohio, United States of America; University of Oldenburg, Germany

## Abstract

Nitric oxide signals through activation of soluble guanylyl cyclase (sGC), a heme-containing heterodimer. NO binds to the heme domain located in the N-terminal part of the β subunit of sGC resulting in increased production of cGMP in the catalytic domain located at the C-terminal part of sGC. Little is known about the mechanism by which the NO signaling is propagated from the receptor domain (heme domain) to the effector domain (catalytic domain), in particular events subsequent to the breakage of the bond between the heme iron and Histidine 105 (H105) of the β subunit. Our modeling of the heme-binding domain as well as previous homologous heme domain structures in different states point to two regions that could be critical for propagation of the NO activation signal. Structure-based mutational analysis of these regions revealed that residues T110 and R116 in the αF helix-β1 strand, and residues I41 and R40 in the αB-αC loop mediate propagation of activation between the heme domain and the catalytic domain. Biochemical analysis of these heme mutants allows refinement of the map of the residues that are critical for heme stability and propagation of the NO/YC-1 activation signal in sGC.

## Introduction

**Figure 1:**
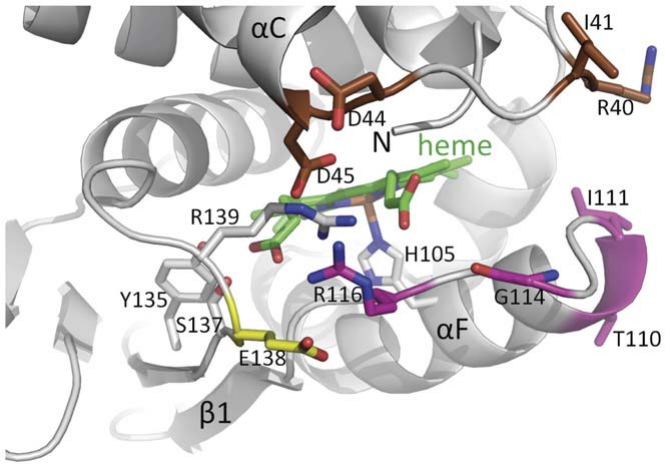
Close-up view of the heme-binding region of a homology model of the heme domain of sGC. The heme (green) and its H105 ligand are depicted. Residues targeted for mutagenesis in the region between the αB-αC helices are colored brown, targeted residues in the αF-β1 strand region are shown in magenta and the flanking E138 residue in yellow. The αC helix, β1 strand, and the αF helix containing the heme-liganding residue H105 are labeled. Residues Y135, S137, and R139 as part of the YxSxR heme binding motif are shown in grey with the residues labeled.

Physiologically, the NO-cGMP signaling pathway is critically involved in vascular homeostasis via smooth muscle relaxation and inhibition of platelet aggregation [1]. Pathophysiologically, dysfunction of this pathway is involved in the development of atherosclerosis and hypertension. Binding of NO to the heme of sGC stimulates several hundred-fold the catalytic production of cGMP from the substrate GTP. A key early event that leads to increased sGC catalytic activity is the NO-mediated breakage of the bond between the heme iron and His105 of the β subunit of sGC (for recent review see [2], [3], [4]). sGC is active as an heterodimer organized in three major domains: the N-terminal domain of the β subunit contains the heme (the N-terminal portion of the α subunit has unknown function, but could interact with the sGC activator YC-1); a central domain formed by the dimerization domain and a coiled-coil helix; a catalytic domain formed by a head-to-tail association of the C-termini of the two subunits, containing the catalytic site and a pseudo-symmetric regulatory site. We and others have solved the structures of (mostly) prokaryotic analog of those domains including the dimerization/PAS fold domain [5], the sGC β1 coiled-coil domain [6], the catalytic domain [7], [8] and the heme domain (HNOX) [9], [10], [11]. However, no crystal structure of the whole sGC molecule exists, greatly impairing our ability to understand the interactions between those different domains, the function of those interactions and the mechanisms by which NO signaling is transmitted to the catalytic domain to increase cGMP formation. Analysis of inactive or active structures of the HNOX domains [11], [12], [13] and molecular simulations [14] indicate that two regions in the heme domain are subjected to major shifts relative to each other upon binding of NO: the αB-αC loop and more noticeably the αF helix-β1 strand loop. Furthermore, the αB-αC loop contains residues D44 and D45, which previously have been shown to be involved in heme incorporation and sGC signal transduction, respectively [15]. These major conformational shifts suggest that these regions of the heme domain could participate in the downstream propagation of the NO binding signal [16]. Our homology modeling studies (Fig. 1) identified a number of partially solvent-exposed residues in those two regions, hence with the potential of being involved in interaction with other sGC subdomains for activation signal propagation. To probe these residues' importance regarding activation, we first conducted an initial screening in COS-7 cells to identify mutants of interest based on their response to NO donors, protoporphyrin IX or YC-1. Four mutants were subsequently purified and their mechanism of activation thoroughly characterized.

## Results

### Homology modeling of the heme domain of the β1 subunit (HNOX) predicts residues involved in NO mediated activation

Figure 1 depicts a close-up view of the homology model of the HNOX heme domain of rat sGC with the two regions of the heme domain that are anticipated to shift upon NO binding relative to each other based on previous structural studies of a homologous heme domain [12]. We predicted that the solvent exposed residues linking the αB-αC helixes and αF-β1 strand region, and flanking E138 residue, could be involved in the interactions with the catalytic domain and/or other sGC subdomains and propagation of the NO signal. This hypothesis was tested by mutational analysis in COS-7 transfected cells.

### Alanine scanning of residues predicted to be involved in propagation of NO activation

For initial screening in a cell-based system, residues in the αF-β1 region (T110, I111, G114, R116, E138) and in the αB-αC loop (I41, R40) were replaced with alanine (A) by site directed mutagenesis. Following transfection in COS-7 cells that do not contain endogenous sGC, cytosolic fractions of WT and mutants were prepared and activity measured under basal (un-stimulated) condition and in the presence of 100 µM Protoporphyrin IX (PPIX), 100 µM YC-1, 100 µM of the NO-donor SNAP, or 100 µM each of YC-1 and SNAP. Table 1 summarizes the basal or stimulated activities of WT and mutant enzymes transiently expressed in COS-7 cells. The levels of expression were similar for WT and mutants, indicating that stability and expression of α and β subunits were not affected by the mutations (Figure S1).

**Table 1:** Basal and stimulated activities of wild type and HNOX mutants in COS-7 cells.

		basal		PPIX, 100 µM		YC-1, 100 µM		SNAP 100 µM		SNAP, 100 µM+YC-1, 100 µM	
		spec. act.	% of WT	spec. act.	fold st	spec. act.	fold st	spec. act.	fold st	spec. act.	fold st
	**WT**	229±7	100	431±42	1.9	1430±63	6.2	3328±102	14.5	3402±370	14.8
**αF-β1 region**	**T110A**	591±60	**254**	800±104	1.4	1691±263	*2.9*	2380±254	*4*	2577±224	*4.4*
	**I111A**	129±7	56	285±18	2.2	851±197	6.6	772±183	*6*	1454±303	11.3
	**G114A**	146±25	64	334±24	2.3	1304±188	8.9	1355±242	9.3	2752±66	18.7
	**R116A**	194±22	84	725±115	**3.7**	694±161	3.6	1245±201	*6.4*	1372±109	*7.1*
	**E138A**	195±12	84	385±5	2.0	2006±163	**10.3**	2904±337	14.9	4423±225	**22.7**
**αB-αC** **loop**	**R40A**	29±4	*13*	68±15	2.3	129±15	4.5	125±14	*4.3*	341±14	11.6
	**I41A**	134±21	58	340±16	2.5	1399±72	**10.5**	3026±348	**22.6**	3465±122	**25.8**

Specific activities (spec. act.) are expressed in pmol. min^−1^. mg^−1^ of cytosolic fraction ± SEM and were measured from 2 to 3 independent transfections. Each assay under each condition was repeated three times and in duplicate. Fold stimulation (fold st) strongly lower or higher than WT is in italic and in bold, respectively. Cytosolic fractions were prepared as described in Material and Methods.

**In the αF-β1 region**, in comparison to the WT and the other mutants, the mutant T110A had an elevated basal activity; mutant R116A was more responsive to PPIX. For WT and mutants other than R116A, PPIX activation was weak, probably because the effect of PPIX is difficult to assess in broken cells. Mutant G114A and E138A were readily activated by YC-1, NO and YC-1+ NO in a manner similar to the WT. I111A had a decreased basal activity and was less responsive to activators in comparison to the WT. These results suggested that residues of the αF region have different roles: T110 could be involved in modulation of basal activity, R116 could be important for heme stabilization while mutation of E138 did not lead to drastic changes suggesting a limited role for this residue under the tested conditions. E138A and I111A, which had only decreased NO response, and G114A with characteristics similar to WT, were not pursued for further studies.

**In the αB-αC loop**, R40 replacement resulted in a drastic decrease not only in basal activity but also in stimulated activity in response to PPIX, YC-1, NO or to the combination of NO+YC-1. Interestingly, replacement of I41, the next residue on the loop, led to opposite characteristics, as I41A was strongly activated by YC-1, NO and to a lesser extent by PPIX and by the combination NO+YC-1. Both mutants of the αB-αC loop were targeted for further studies.

To determine the involvement of the above residues in modulation of basal activity, in heme stability and understand the mechanisms of propagation of NO and YC-1 activation, several of the mutants were purified for spectral and kinetics analyses.

### Purification and heme content of WT and mutants

**Figure 2:**
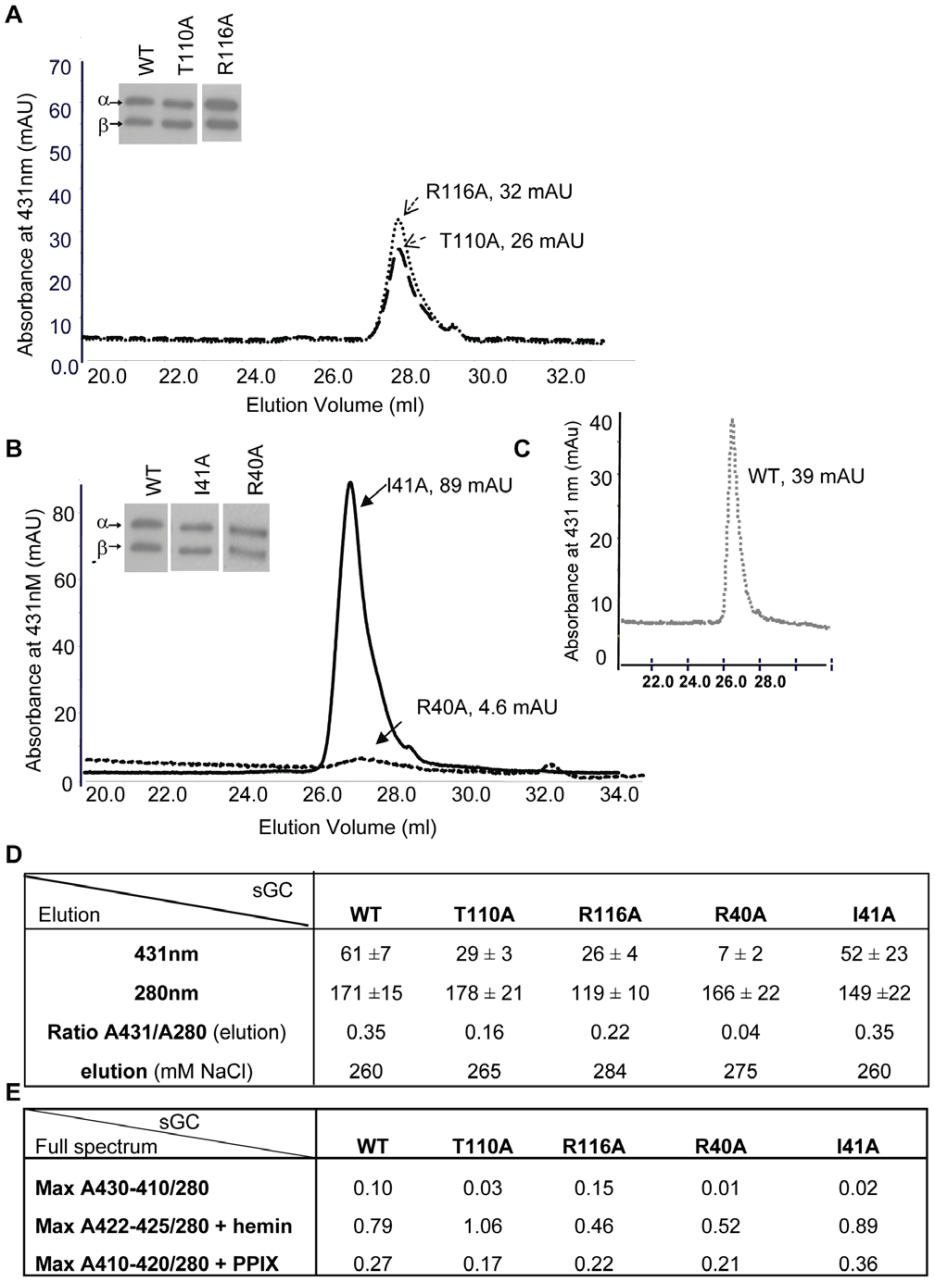
Purification of Wild Type and mutants. 2A–B: representative spectra of purification of mutants at 431 nm, the expected absorbance for the heme-containing enzyme. All mutants had similar levels of expression as assessed by immunoblot with antibodies against α and β subunit of sGC after electrophoresis of ∼0.5 µg of semi-purified protein on a 7.5% Tris-HCl gel (insets). 2C: representative elution profile of purification of WT. 2D: Table of purification with ratio of heme-containing sGC (431 nm) over protein total concentration (280 nm) from the elution, measured as described in *Material and Methods*; values are expressed in absorbance unit (mAU). Elution as a function of salt concentration is indicated (NaCl, mM). Measurement at 393 nm to estimate oxidized heme-containing sGC was not significantly different between WT and mutants (not shown). 2E: Table of full spectrum values. UV-vis was recorded as described in Material and Methods (see also Figure S3) and the ratio of maximum absorption for WT and mutants between 410 and 430 nm over maximum absorption at 280 nm was calculated in the absence or presence of hemin (5 µM) and PPIX (5 µM) after heme reduction with 5 mM DTT. Max: Maxima. Each mutant was purified at least three times (±S.E.M.) and WT was purified six times (± S.E.M.). UV-vis collection of the various Soret bands for WT and mutants are shown in Figure S3 and Figure S4.

Mutant and WT were expressed and semi-purified as described in *Material and Methods*. At elution, absorbance was monitored at 431 nm, which is the expected absorbance for non-stimulated heme-containing sGC (Fig. 2 A–C). Absorbance was also measured at 280 nm for total protein concentration. The ratio of total protein to heme bound protein of the WT and mutants was initially used to estimate heme content (Fig. 2D) with the caveat that WT and mutants were partially purified (Coomassie staining, Figure S2). For the αF-β1 loop residues, T110A and R116A retain the heme only partially, as reflected in the ratio A431/A280 nm when compared to WT (Fig. 2D). In the αB-αC loop, the replacement of R40 and I41 led to opposite A431/A280 values (Fig. 2B and 2D). R40A was mostly in apo form with an A431/A280 ratio of 0.04 (Fig. 2D and Figure S3). Western blot analysis (insets) showed that for all purified mutants both subunits were equally expressed, most likely reflecting a stable heterodimer. Overall, the WT and mutants have lower values of heme content (expected value of 1). To determine whether this was due to poor heme loading, we measured the full spectrum (600 to 240 nm) in the absence or presence of hemin. The full spectrum indicated lower maxima absorbance ratio A430/A280 (Fig. 2E and Figure S3 and Figure S4). Interestingly, the mutation R116A shifted the heme Soret band from 431 nm to 410 nm. Values of heme content of 1 for T110A and close to 1 for WT and I41A were obtained upon addition of 5 µM hemin (Fig. 2E). On the other hand, mutant R40A and R116A were only 50% reconstituted with hemin. In summary, mutant R40A and R116A have partially lost affinity for the heme whereas mutants T110A and I41A have decreased stability for the heme compared to WT.

### Basal and stimulated activities of purified WT and mutants

**Figure 3:**
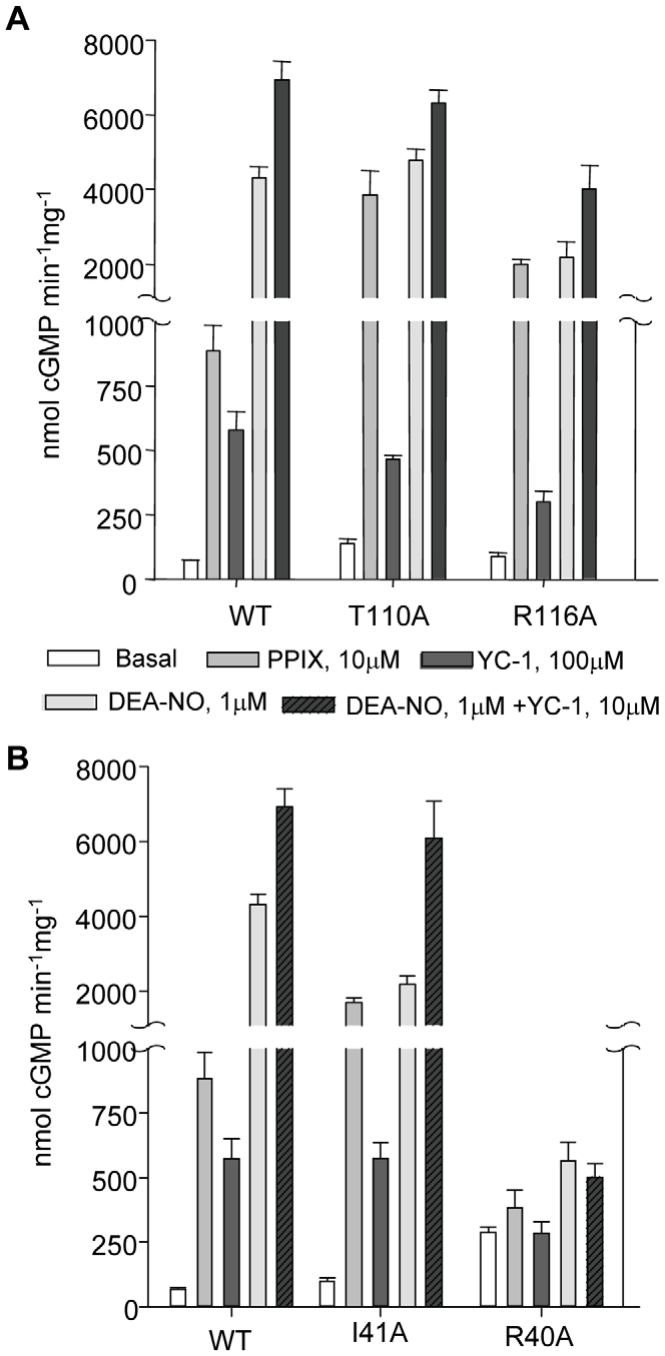
Basal and stimulated activities of purified WT and mutants. 3A: In the αF-β1 region, basal activity of α1/β1T110A as well as PPIX activation of α1/β1T110A and α1/β1R116A were significantly higher than WT. 3B: In the αB-αC loop, mutant α1/β1I41A exhibits a significantly higher response to PPIX and significantly lower response to DEA-NO compared to WT, yet sensitivity to DEA-NO +YC-1 was comparable to WT. α1/β1R40A lost the ability to respond to any activators. Measurements of activities were done at least three times on 50 ng of two to three independently semi-purified WT and mutants with each assay done in duplicate under each condition. Results are expressed in nmol cGMP. mg^−1^.min^−1^ ± S.E.M. Values and fold stimulation are provided in Table S1.

Compared to basal activity, the purified WT was stimulated 12-fold by 10 µM PPIX, 8-fold by 100 µM YC-1, 59-fold by 1 µM DEA-NO and 90-fold by 10 µM YC-1+10 µM DEA-NO (Fig. 3 and Table S1).

**In the helix αF-β1 region** (Fig. 3A), mutant T110A has several interesting characteristics; first, as in the initial screening in COS-7 cells, the purified mutant showed a 2-fold increase of its basal activity compared to WT (Table S1). Second, it was stimulated 28-fold by PPIX (vs. 12-fold for the WT) while its response to NO, YC-1 or NO+YC-1 was similar to WT but decreased if the fold-stimulation compared to basal was taken into account. The strong stimulation by PPIX could be explained by the existence of an apo form of the mutant, in addition to the heme-bound form as the T110A A430/A280 ratio was low compared to WT. Yet, reconstitution with PPIX expressed as function of the ratio of A410 nm (expected absorbance for PPIX occupying the heme domain) over A280 nm indicated a lower value for T110A compared to WT (Fig. 2E and Figure S4). This suggested a higher PPIX activation in comparison to WT (Table S1). These characteristics, including high response to PPIX but with lower heme content were also seen in mutant R116A. R116A, unlike T110A, does not display high basal activity and had lower response to DEA-NO. Overall, T110A seems to be more sensitive to NO and PPIX activation (Table S1) than WT, considering its low A430/A280 and A410/A280 ratios. As such, the response to PPIX and NO was further studied in mutant T110A by conducting concentration-response curves (see below).

**In the αB-αC loop** (Fig. 3B), mutant R40A did not respond to NO as expected for a heme-depleted mutant but was also unresponsive to YC-1, to the combination of DEA-NO+YC-1, and to PPIX indicating that this mutation drastically affects not only the heme stability but the overall catalytic activity. As R40A expression of both subunits was not affected, these results support the idea that the stability of the heme domain is critical for any type of activity including basal catalytic activity. In addition, R40A was only partially reconstituted by hemin or PPIX suggesting a profound alteration of the heme binding pocket (Fig. 2E). In sharp contrast, mutant I41A, which displays a low A430/A280 ratio on the full spectrum (Fig. 2E and Figure S3 and Figure S4), was highly stimulated by PPIX (>16-fold), which was confirmed by the highest PPIX reconstitution value (A410/A280 ratio, Fig. 2E). I41A had a decreased response to DEA-NO (in comparison to WT), and DEA-NO+YC-1, while response to YC-1 was similar to WT. To further investigate mutant I41A, dose response curves to PPIX and DEA-NO were conducted in parallel with WT and mutant T110A.

### Dose response curves to NO and PPIX of WT, T110A and I41A

**Figure 4:**
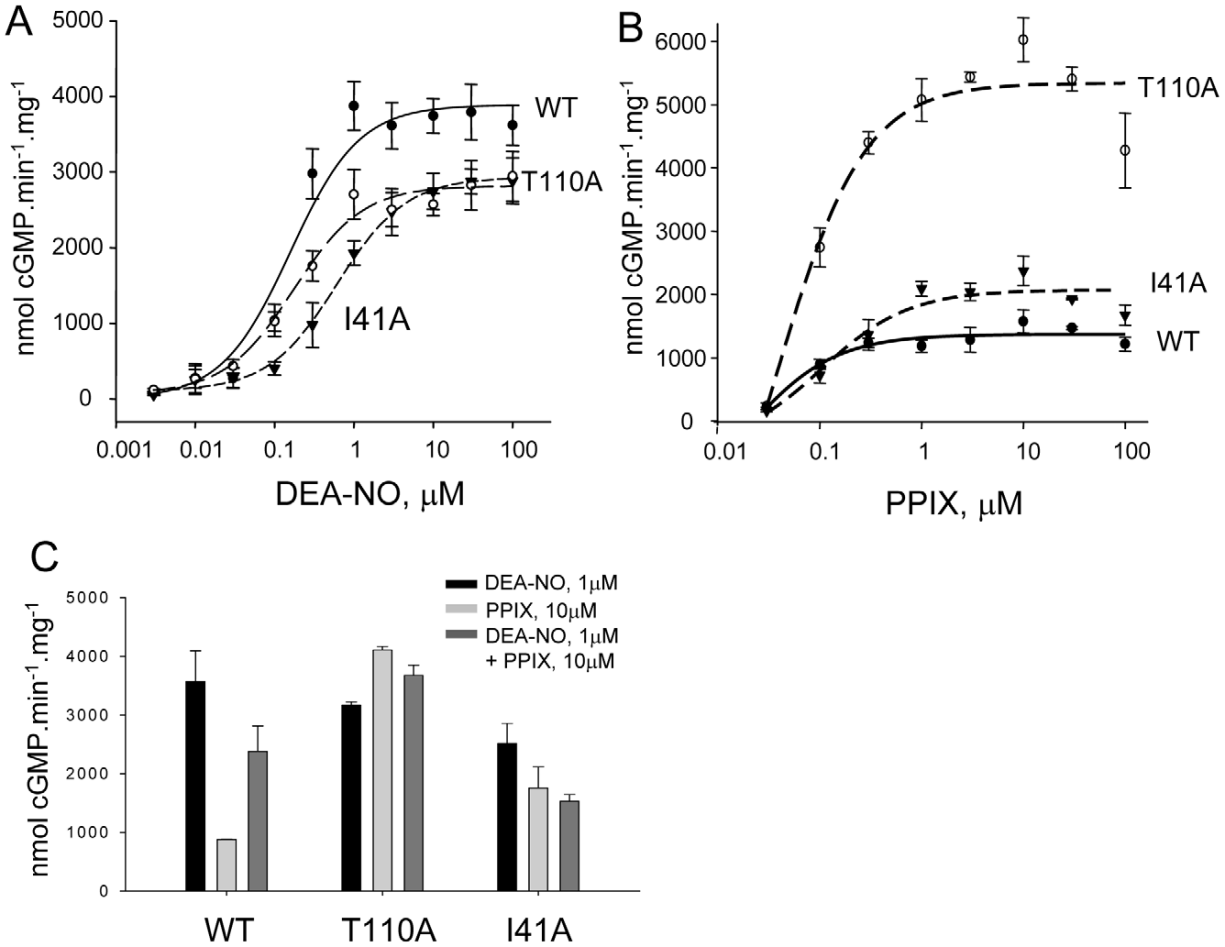
Response of WT, α1/β1T110A and α1/β1I41A to DEA-NO, PPIX and both. 4A: DEA-NO concentration-response curve of purified WT and mutants. cGMP production was measured as a function of increasing concentrations of DEA-NO. 4B: PPIX concentration-response curves of purified WT and mutants. cGMP production was measured as a function of increasing concentrations of PPIX. 4C: Comparison of WT and mutants' response to DEA-NO (1 µM), PPIX (10 µM) and DEA-NO (1 µM)+PPIX (10 µM). Results are expressed in nmol cGMP. mg^−1^.min^−1^ ± S.E.M. Typically, 50 ng of semi-purified enzyme was used per assay tube. Each experiment, carried out in duplicate, was repeated independently two or three times with two separate purified preparations. For the concentration-response curves, EC_50_ and maximal velocity were determined using Sigmaplot software (version 11.0).

In response to increasing concentrations of DEA-NO (Fig. 4A), the maximal velocity (V_max_) was slightly higher for WT (3890.1±167.5 nmol.min^−1^.mg^−1^) than for T110A and I41A (2812.4±77.6 and 2915.1±54.3 nmol.min^−1^.mg^−1^, respectively) whereas the EC_50_ for DEA-NO of WT and T110A was similar (0.15±0.04 µM vs. 0.16±0.02 µM) but higher in I41A (0.50±0.05 µM). The slight decrease in Vmax at saturating concentrations of DEA-NO together with unchanged EC_50_ (compared to WT) suggest that T110 is involved in the efficacy of stimulation by DEA-NO rather than the affinity of NO for the heme. On the other hand, the mutation I41A negatively affects efficacy and affinity of DEA-NO. For T110A and I41A, Vmax is reached at higher concentration of DEA-NO, which is probably due to heme depletion as both mutants show a decrease in the ratio of maxima A430/280 in the full spectrum measurement (Fig. 2E and Figure S3). Conversely, T110A and to a lesser extent I41A were more responsive than WT to PPIX (Fig. 4B); T110A exhibited a V_max_ 4-fold and 2.5-fold higher than WT and I41A, respectively (5369±344 nmol.min^−1^.mg^−1^ vs. 1325±80 and 2018±138 nmol.min^−1^.mg^−1^). Higher V_max_ for I41A in response to PPIX correlated with a higher PPIX reconstitution compared to WT (Fig. 2E). The high activation by PPIX of T110A could partially be explained by a mixture of apo and heme-bound forms for T110A. However, the mixture of apo and heme-bound forms for T110A does not explain the only slightly reduced response to DEA-NO of T110A compared to WT and the high activation by PPIX (considering the low PPIX reconstitution value). As such, T110A appears more responsive than WT to DEA-NO and PPIX stimulation. To better understand these observations, activation in presence of both PPIX (10 µM) and DEA-NO (1 µM) was assayed. Fig. 4C indicates that at these concentrations, the effect of the two activators is not additive for the WT and mutants; on the contrary, the combined DEA-NO/PPIX-stimulated activity is less than the NO-stimulated activity, which could be explained by the fact that 10 µM PPIX, by displacing the heme, lowers the NO-dependent stimulation. However, activities of T110A are similarly stimulated in response to DEA-NO or PPIX or both, which could suggest that two populations (apo and heme-bound form) are present but that the DEA-NO efficacy and PPIX activation are enhanced in T110A.

## Discussion

In spite of studies that identified residues critical for the heme stability and binding [17], [18] and that described inhibitory interactions between the heme domain and the catalytic domain [19], [20], the major challenge remains to understand the mechanism of propagation of the NO signal between the receptor-heme domain (at the NH_2_-terminal of the sGC molecule) to the effector-catalytic domain (at the C-terminus of sGC). It is accepted that breaking of the His-iron heme bond upon binding of NO is a key step in inducing conformational changes leading to increase catalysis in the catalytic domain [21]. In parallel, it is clear that large shifts in regions of the heme domain occur during activation. How these shifts lead to increased catalytic activity is similarly unresolved.

To address these fundamental questions, we first carried out homology modeling of the rat sGC heme domain to identify solvent-exposed residues in the regions that exhibit the largest shifts, the αF helix-β1 strand and the flanking αB-αC loop, as recently studied in our structure of the sGC activator BAY 58–2667 bound to Ns HNOX [22] and in our structure-function analysis of residues involved in NO propagation downstream of the His-iron breakage [16]. As such, these two regions could be the missing link to propagate activation, i.e. be directly involved in interaction between the heme domain and the catalytic domains as proposed by others [20] or induce conformational changes, transmitted by the intermediary domains (PAS and coiled-coil domains).

In the αF helix region, replacement of residue R116 with Ala led to decreased activation by YC-1, DEA-NO and YC-1+DEA-NO, as expected if this region is involved in propagation of activation. Interestingly, the full UV-Vis spectrum of the semi-purified R116A revealed that the Soret band absorption maximum was blue-shifted from 431 nm to 410 nm, which could indicate a weakening and breakage of the His-iron bond in this mutant [23]. In addition, the reconstitution with hemin was only partial compared to WT or T110A of the αF helix (Fig. 2E). These data suggest that the replacement of residue R116 with Ala not only affect the spectral properties of the heme group but also affects heme stability and affinity. However, in the same region the replacement of T110 with Ala only slightly reduced the V_max_ of the DEA-NO response curves. Interestingly, T110A also displayed a 2-fold increase in basal activity compared to WT, which partially supports the idea that under basal conditions, the heme domain has an inhibitory effect on the catalytic domain [19], as replacement with Ala could partially relieve the inhibition. Furthermore, considering the low heme content of T110A, the response of T110A to DEA-NO was high compared to WT while EC_50_ was similar. We speculate that T110 is a key residue for interactions between the heme and catalytic domains. This supports our most recent structural analysis with the compound BAY58–2667 that predicted the largest shift resided in the αF carboxy-terminus, which includes T110 [22]. Those results suggest that T110 is a critical residue for basal activity and NO activation by “loosening” a potential inhibitory interaction between the catalytic and heme domains. This will also explain the significant higher activation of T110A by PPIX over WT despite a lower PPIX reconstitution, in addition to the fact that the higher response to PPIX is also partially due to the T110A apo form, as observed previously with the R139 mutant [17]. Thus, T110 and to a lesser extent R116 appear to be crucial residues for heme stability/affinity as shown by the lower heme content and increased PPIX response when replaced with Ala. The function of these residues (T110, R116) in sGC is different from other residues of the same αF helix, in particular D102 and F120 [16]. D102 and F120 are also involved in propagation of the NO signal yet unlike T110 or R116, D102 is predicted to interact with the backbone of F120 and with H105 via a molecule of water. As a result, in mutant D102N the breakage of the His-iron bond and the activation by NO were uncoupled [16]. These contrasting phenotypes of mutants introduced in the same helix underscore the fine-tuned mechanism of propagation of the NO signal from the receptor heme domain to the effector catalytic domain of sGC.

Interestingly, replacement of R40 and I41 with Ala in the αB-αC loop led to opposite phenotype suggesting different roles in propagation of activation. R40A was predicted to be located in the surface contact region between heme and catalytic domains (Fig. 1 and [22]), and to act as a potential switch for activation as described for D45 [15]. Instead, R40A led to a heme-depleted enzyme unresponsive to the activators tested here. This is reminiscent of the phenotypes of mutant D44A [15]; a potential reason for depletion of the heme could be due to the introduction of Ala in place of R40 (and of D44 in the study cited above), resulting in a highly hydrophobic region (L38-V39-**A40**-I41-I42). Such a hydrophobic region could potentially be more prone to adopting an alternate conformation, as there are no longer hydrophilic or charged residues that can provide conformational specificity of this region. With this region being in close proximity to the N-terminus of the heme domain which is involved in heme propionate group binding [11], we anticipate that shifts in this L38-I42 region upon mutating R40A will affect the position of the N-terminal region and thus heme binding. Indeed, the reconstitution with hemin was only partial in this mutant, as was the PPIX reconstitution (Fig. 2E). On the other hand, I41A mutation probably reduces hydrophobicity of this region, hence the heme could be repleted in I41A mutant and the reconstitution with PPIX was higher than WT. The decreased response of this mutant to DEA-NO and higher basal activity suggest that I41 is important for activation. I41 is also probably involved in heme stability as the response of the mutant to PPIX is higher, in agreement with higher PPIX reconstitution value and a lower A430/280 ratio (Fig. 2E).

In summary, our studies and results in probing of the αF helix and αB-αC loop regions of the heme sGC domain indicate their involvement in propagation of NO-dependent and independent activation and/or impact on heme stability.

## Materials and Methods

### Molecular modeling

Molecular modeling of the heme domain of sGCβ1 is carried out as described previously [12]. Molecular figure was made using PYMOL (www.pymol.org).

### Reagents

[α-^32^P]-GTP is from PerkinElmer (MA, USA). DMEM from ATCC (VA, USA) and antibiotic/antimycotic from Mediatech, Inc. (VA, USA). The expressfect transfection reagent is from Denville Scientific Inc. (NJ, USA) and SF900II SFM medium, FBS and Gentamycin from Invitrogen. SNAP from EMD chemicals (NJ, USA), DEA-NO from Axxora LLC (CA, USA). β sGC antibody are from Cayman chemicals (MI, USA). 7.5% Tris-HCl gels are from Bio-Rad Laboratories (CA, USA). All the other reagents were from Sigma.

### Cell cultures

COS-7 cells from ATCC (CRL-1651, VA, USA) were grown in DMEM supplemented with 10% fetal bovine serum (FBS) and 1% antibiotic/antimycotic (penicillin (100 IU/ml), streptomycin (100 µg/ml) and Amphotericin B (0.25 µg/ml)). Sf21 cells were seeded at 0.5×10^6^ cells/ml in 2 L of SF900II SFM medium containing gentamycin (0.1 mg/ml) and 5% heat inactivated FBS at 28°C with constant shaking.

### Mutation, transfection in COS-7 cells and cytosolic preparation of sGC

α1 and β1 subunits of rat cDNA subcloned in pCMV5 vector [24] were mutated using Quikchange Site-Directed Mutagenesis Kit (Agilent Technologies, CA, USA) and confirmed by sequencing. COS-7 cells were transiently transfected for 48 h with α1 and β1 WT or β1 mutant cDNA as described before[25]. After 48 h, transfected COS-7 cells were washed in cold phosphate buffered saline (PBS) and scraped in homogenization buffer (50 mM HEPES, pH 8.0, 150 mM NaCl, 1 mM DTT, protease inhibitors). Cytosol was prepared by sonication and centrifugation at 16000 rpm for 10 min at 4°C to remove the membrane fraction and cell debris. Expression levels of sGC WT and mutants were assessed by immunoblot with antibodies against α and β after electrophoresis of 8–10 µg of cytosolic protein on a 7.5% Tris -HCl gel.

### Construction and purification of mutant in insect Sf21 cells/baculovirus system

Mutations were introduced in the β1 subunit with an His-tag at the C-terminus and cloned in the baculovirus transfer vector pbacPAK8 as described previously [25]. Recombinant baculovirus were produced in Sf21 cells (Invitrogen, CA, USA) by co-transfection of the pbacPAK8 construct with the linear BacPak6 viral DNA (Clontech, CA, USA). Sf21 cells were co-infected with α1- and β1-His_tag_ WT or mutant containing viruses and grown for 48 hours. The recombinant protein was purified in two steps using a cobalt column followed by Mono Q anion exchange column, as previously described [25]. Chromatographic separation was performed at a flow rate of 0.5 mL/min. The elution pattern at 431, 280 and 393 nm was recorded using Unicorn program of the GE ÄKTA HPLC/FPLC purifier. The level of purification was directly estimated by the ratio OD431 nm/OD280 nm simultaneously measured (similar background noise) by the GE ÄKTA FPLC UV-900 detector (Table Figure 2D values). Each enzyme was purified three (mutants) to six (WT) times. The fractions that showed a peak at 431 nm were collected and snap frozen in 10% glycerol for storage at −80°C.

### UV-visible spectroscopy

UV-visible spectroscopy was carried out using a Shimadzu 2450 double monochromator spectrophotometer on the pooled fractions collected above. Full spectra from 600 nm to 240 nm were collected for semi-purified wild type and each mutant with 72–80 uL (∼600 ng) enzyme in a quartz microcuvette. Heme reconstitution was carried out with both hemin and protoporphyrin IX (PPIX). 40–72 µL (∼575 ng) of enzyme was incubated with 5 µM hemin or 5 µM PPIX in the presence of 5 mM DTT (for heme reduction) for 15 minutes at room temperature (25°C). The reference cuvette contained the equivalent volume of 50 mM HEPES, pH 8.0, 5 µM hemin or PPIX and 5 mM DTT also incubated for 15 minutes. A new reference was prepared with each new sample. The full spectrum from 600 nm to 240 nm was collected. The heme content versus protein was compared as a ratio of absorbencies at 280 nm and 431 nm corrected for baseline for purified and hemin-reconstituted. The heme Soret peak for PPIX was shifted to approximately 410 nm, so the ratio of absorbencies at 280 nm and 410 nm corrected for baseline was determined for the PPIX-reconstituted samples for comparison.

### sGC activity assay

sGC activity was measured by the conversion of [α-^32^P]cGMP from [α-^32^P]GTP as previously described [25]. For DEA-NO and PPIX concentration-response curves of purified WT and mutants, sGC activity was measured in duplicate and in the presence of ten different concentrations of DEA-NO (ranging from 0.003 to 100 µM) and eight different concentrations of PPIX (ranging from 0.03 to 100 µM). The EC_50_ values were calculated from the concentration-response curves that were established from three independent experiments and corresponded to the concentration of DEA-NO or PPIX that half-maximally activates the enzymes.

### Statistical analysis

The results are expressed as mean ± S.E.M from the data derived from [α-^32^P]GTP assay. Activity measurements in cytosol of COS-7 cells were repeated three times, each in duplicates and from two to three independent transfections. For purified enzyme assays, two to three independent enzyme semi-purified preparations were used and assayed four times under each condition and in duplicate. Student's *t* test was used for statistical comparison between groups and conditions with Sigmaplot version 11.0 software (Systat software, San Jose, CA). *P*<0.05 was considered statistically significant.

## Supporting Information

Figure S1 Western blot of WT and HNOX mutants transiently expressed in COS-7 cells. Western blot analysis with antibodies against the α and β subunits of WT and HNOX mutants transiently expressed (48 h) in COS-7 cells. 10 µg of cytosolic fraction were electrophorated on 8% SDS-gel. A and B are two separate transfections.
(PDF)

Figure S2 Coomassie blue staining of semi-purified WT and HNOX mutants. Approximately 5 µg of each WT and mutants were electrophorated on an SDS-gel under reducing, denaturing conditions and stained with Coomassie.
(PDF)

Figure S3 Spectra of absorbance of semi-purified WT and mutants. UV-Vis (240–600 nm) was collected as described under Material and Methods. Arrow indicates the maximum peak between 410 and 430 nm. No peak was detectable for R40A.
(PDF)

Figure S4 Spectra of absorbance of semi-purified WT and mutants in the absence or presence of 5 mM hemin and PPIX. Heme was reduced with 5 mM DTT. UV-Vis (240–600 nm, only 350–500 nm shown) was collected as described under Material and Methods.
(PDF)

Table S1 Comparison between purified WT and mutants of basal and stimulated activities. The values correspond to Fig. 3 and include the fold stimulation of each activator compared to basal activity for WT and mutants and percentage of basal activity of the mutants compared to WT. Specific activity is expressed in nmol.min−1.mg−1± SEM. Stim.: stimulation. *: The higher basal activity of R40A is explained by a higher amount of sGC in the fraction used, i.e. the fraction eluted with high salt (∼500 mM) with some absorbance at 431 nm as well.
(PDF)
