# Supplementary material for: Identification of Residues in the Heme Domain of Soluble Guanylyl Cyclase that are Important for Basal and Stimulated Catalytic Activity
Source: PLoS One. 2011 Nov 9;6(11):e26976. doi: 10.1371/journal.pone.0026976 (PMC3212528; doi:10.1371/journal.pone.0026976)
Supplement: Figure S1 — Western blot of WT and HNOX mutants transiently expressed in COS-7 cells. Western blot analysis with antibodies against the α and β subunits of WT and HNOX mutants transiently expressed (48 h) in COS-7 cells. 10 µg of cytosolic fraction were electrophorated on 8% SDS-gel. A and B are two separate transfections. (PDF) [file pone.0026976.s001.pdf]

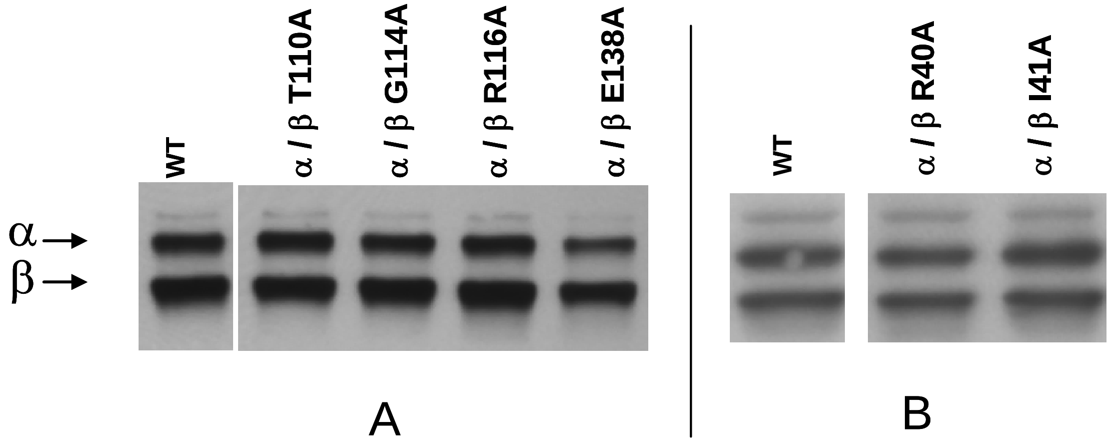

Figure S1: Western blot analysis with antibodies against the α and β subunits of WT and HNOX mutants transiently expressed (48 h) in COS-7 cells. 10μg of cytosolic fraction were electrophorated on 8% SDS-gel. A and B are two separate transfections
