# Supplementary material for: Identification of Residues in the Heme Domain of Soluble Guanylyl Cyclase that are Important for Basal and Stimulated Catalytic Activity
Source: PLoS One. 2011 Nov 9;6(11):e26976. doi: 10.1371/journal.pone.0026976 (PMC3212528; doi:10.1371/journal.pone.0026976)
Supplement: Figure S2 — Coomassie blue staining of semi-purified WT and HNOX mutants. Approximately 5 µg of each WT and mutants were electrophorated on an SDS-gel under reducing, denaturing conditions and stained with Coomassie. (PDF) [file pone.0026976.s002.pdf]

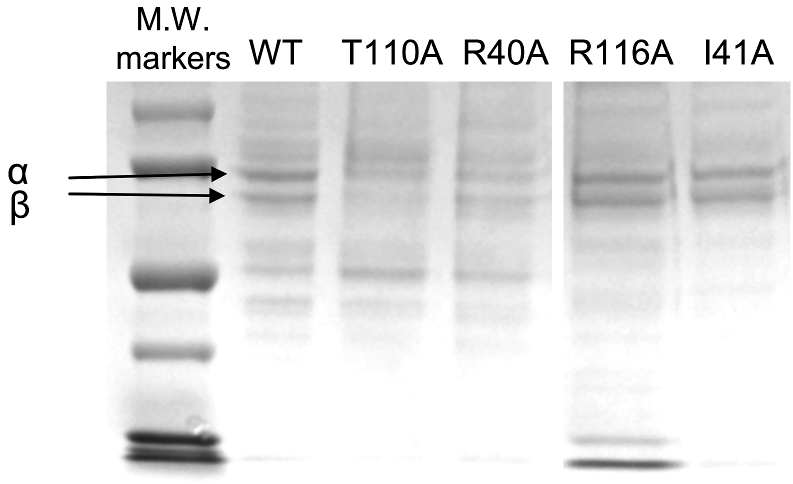

<u>Figure S2</u>: Coomassie blue staining of semi-purified WT and HNOX mutants. Approximately 5 µg of each WT and mutants were electrophorated on an SDS-gel under reducing, denaturing conditions and stained with Coomassie.
