# Supplementary material for: Identification of Residues in the Heme Domain of Soluble Guanylyl Cyclase that are Important for Basal and Stimulated Catalytic Activity
Source: PLoS One. 2011 Nov 9;6(11):e26976. doi: 10.1371/journal.pone.0026976 (PMC3212528; doi:10.1371/journal.pone.0026976)
Supplement: Figure S3 — Spectra of absorbance of semi-purified WT and mutants. UV-Vis (240–600 nm) was collected as described under Material and Methods. Arrow indicates the maximum peak between 410 and 430 nm. No peak was detectable for R40A. (PDF) [file pone.0026976.s003.pdf]

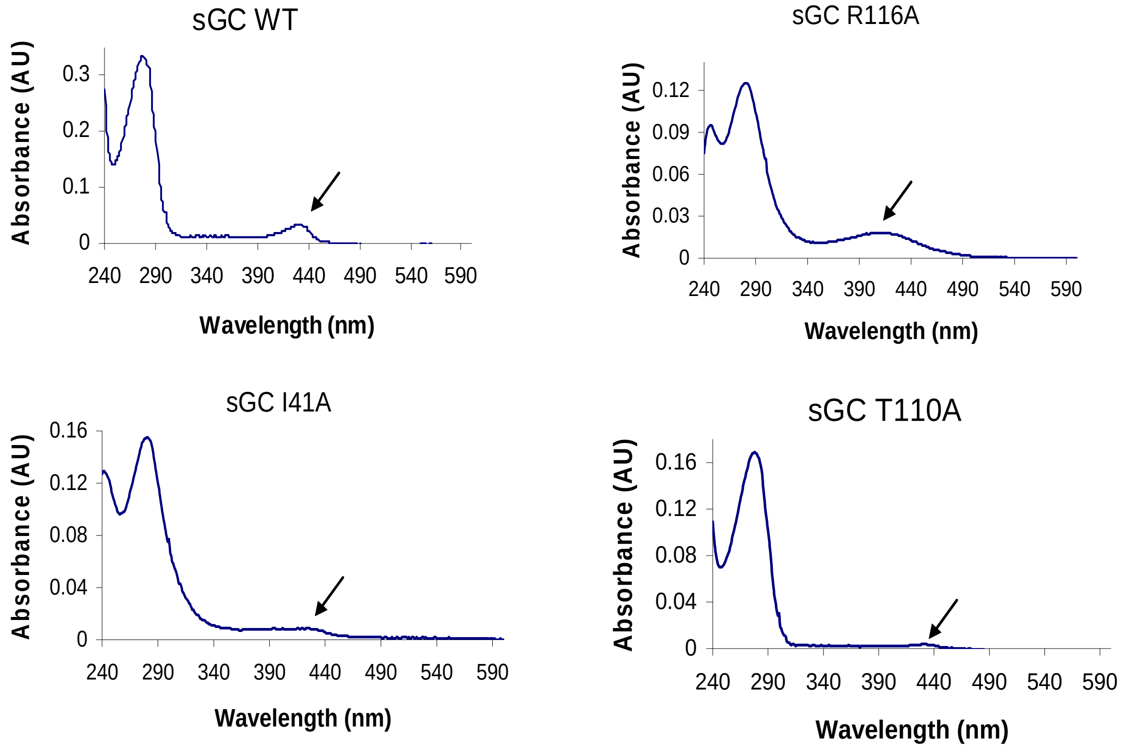


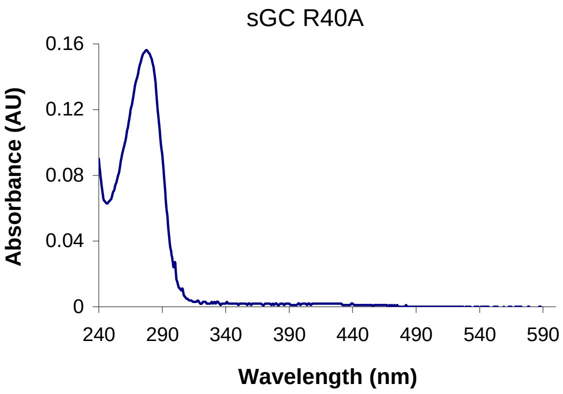


Figure S3: Spectra of absorbance (240-600nm of semi-purified WT and mutants. UV-Vis was collected as described under Material and Methods. Arrow indicates the maximum peak between 410 and 430 nm and the corresponding maximum value. No peak was detectable for R40A.
