# Supplementary material for: Identification of Residues in the Heme Domain of Soluble Guanylyl Cyclase that are Important for Basal and Stimulated Catalytic Activity
Source: PLoS One. 2011 Nov 9;6(11):e26976. doi: 10.1371/journal.pone.0026976 (PMC3212528; doi:10.1371/journal.pone.0026976)
Supplement: Figure S4 — Spectra of absorbance of semi-purified WT and mutants in the absence or presence of 5 mM hemin and PPIX. Heme was reduced with 5 mM DTT. UV-Vis (240–600 nm, only 350–500 nm shown) was collected as described under Material and Methods. (PDF) [file pone.0026976.s004.pdf]

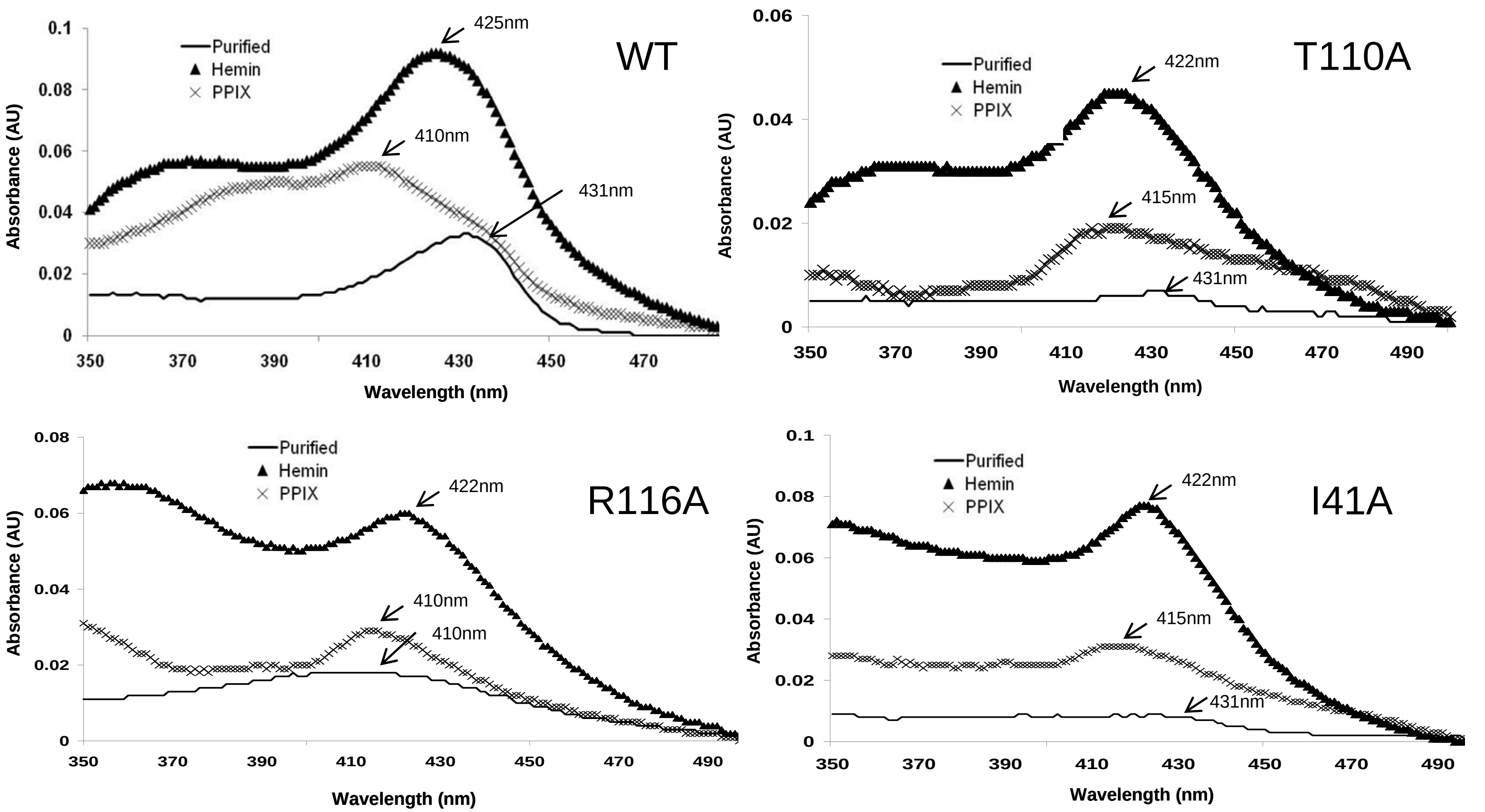

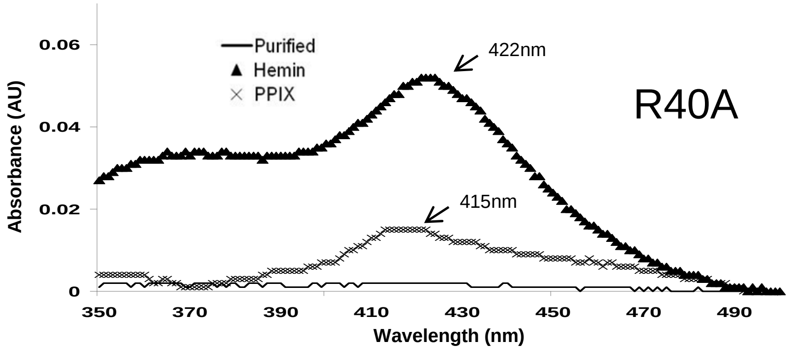

Figure S4: Spectra of absorbance (240-600nm, only 350-500 nm shown) of semi-purified WT and mutants in the absence or presence of 5μM hemin and PPIX after heme reduction with 5mM DTT. UV-Vis was collected as described under Material and Methods.
