# Supplementary material for: Identification of Residues in the Heme Domain of Soluble Guanylyl Cyclase that are Important for Basal and Stimulated Catalytic Activity
Source: PLoS One. 2011 Nov 9;6(11):e26976. doi: 10.1371/journal.pone.0026976 (PMC3212528; doi:10.1371/journal.pone.0026976)
Supplement: Table S1 — Comparison between purified WT and mutants of basal and stimulated activities. The values correspond to Fig. 3 and include the fold stimulation of each activator compared to basal activity for WT and mutants and percentage of basal activity of the mutants compared to WT. Specific activity is expressed in nmol.min−1.mg−1± SEM. Stim.: stimulation. *: The higher basal activity of R40A is explained by a higher amount of sGC in the fraction used, i.e. the fraction eluted with high salt (∼500 mM) with some absorbance at 431 nm as well. (PDF) [file pone.0026976.s005.pdf]

Table S1: Comparison between purified WT and mutants of basal and stimulated activities. The values correspond to Fig.3 and include the fold stimulation of each activator compared to basal activity for WT and mutants and percentage of basal activity of the mutants compared to WT. Specific activity is expressed in $nmol.min^{-1}.mg^{-1}$ ± SEM. Stim.: stimulation. *: The higher basal activity of R40A is explained by a higher amount of sGC in the fraction used, i.e. the fraction eluted with high salt (~500mM) with some absorbance at 431nm as well.

| | BASAL | % WT | DEA-NO, 1 μM | fold stim. | YC-1, 100 μM | fold stim. | DEA-NO, 1 μM + YC-1, 10 μM | fold stim. | PPIX, 10 μM | fold stim. |
|---|---|---|---|---|---|---|---|---|---|---|
| WT | 73.1 ± 2.0 | 100 | 4310.0 ± 298.8 | 59.0 | 561.5 ± 75.2 | 7.9 | 6566.9 ± 208.2 | 89.8 | 886.1 ± 99.6 | 12.1 |
| T110A | 138.0 ± 18.8 | 194 | 4769.2 ± 327.3 | 34.5 | 465.2 ± 13.9 | 3.4 | 5548.9 ± 803.1 | 40.2 | 3843.8 ± 658.2 | 27.9 |
| R116A | 87.5 ± 14.8 | 119 | 2181.9 ± 424.6 | 24.9 | 299.0 ± 43.0 | 3.4 | 4007.0 ± 641.0 | 45.8 | 2003.0 ± 126.9 | 22.9 |
| I41A | 103.8 ± 9.4 | 140 | 2187.2 ± 218.6 | 21.1 | 578.5 ± 59.6 | 5.6 | 5973.4 ± 705.2 | 57.4 | 1700.4 ± 122.7 | 16.3 |
| R40A * | 293.2 ± 15.4 | ----- | 369.5 ± 70.2 | 1.9 | 289.3 ± 39.9 | 1 | 515.3 ± 43.7 | 1.7 | 386.3 ± 66.4 | 1.3 |
